# Dimethyl *cis*-2-methyl-3-*p*-tolyl­isoxazolidine-4,5-dicarboxyl­ate

**DOI:** 10.1107/S1600536809009350

**Published:** 2009-03-19

**Authors:** Mustafa Odabaşoğlu, Hamdi Özkan, Yılmaz Yıldırır, Orhan Büyükgüngör

**Affiliations:** a Pamukkale University, Denizli Higher Vocational School, Chemistry Program, Tr-20159 Kınıklı, Denizli, Turkey; b Department of Chemistry, Faculty of Arts and Science, Gazi University, Ankara, Turkey; c Department of Physics, Faculty of Arts and Science, Ondokuz Mayıs University, TR-55139 Kurupelit, Samsun, Turkey

## Abstract

In the mol­ecule of the title compound, C_15_H_19_NO_5_, the isoxazole ring adopts an envelope conformation. In the crystal structure, weak inter­molecular C—H⋯O and C—H⋯N hydrogen bonds link the mol­ecules, in which they may be effective in the stabilization of the structure.

## Related literature

For general background, see: Tufariello (1984 ▶); Villamena & Zweier (2004 ▶); Halliwell (2001*a* ▶,*b* ▶); Zweier & Talukder (2006 ▶); Janzen (1971 ▶, 1980 ▶); Janzen & Haire (1990 ▶); Villamena *et al.* (2007 ▶); Floyd & Hensley (2000 ▶); Inanami & Kuwabara (1995 ▶); Becker *et al.* (2002 ▶). For bond-length data, see: Allen *et al.* (1987 ▶). For the preparation of *N*-Methyl-C-(-4-methylphenyl) nitrone, used in the synthesis, see: Heaney *et al.* (2001 ▶). For 1,3-dipolar cycloaddition of nitrones and alkenes, see: Confalone & Huie (1988 ▶); Torssell (1988 ▶); Frederickson (1997 ▶); Gothelf & Jorgensen (1998 ▶).
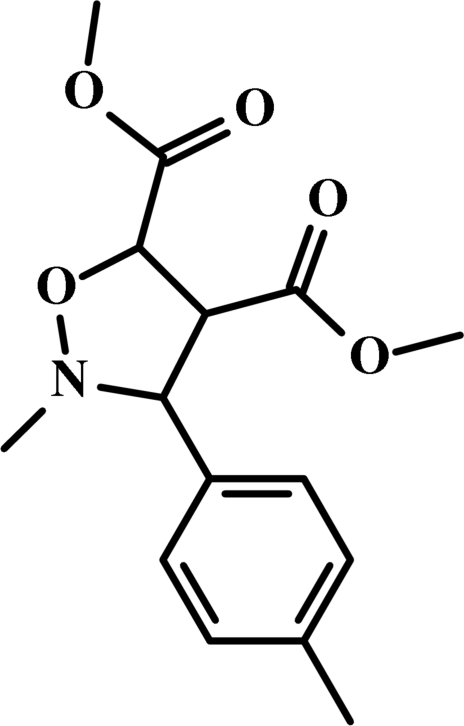


## Experimental

### 

#### Crystal data

C_15_H_19_NO_5_
*M* *_r_* = 293.31
Orthorhombic, 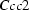

*a* = 15.3832 (7) Å
*b* = 19.7959 (8) Å
*c* = 9.9612 (3) Å
*V* = 3033.4 (2) Å^3^
*Z* = 8
Mo *K*α radiation
μ = 0.10 mm^−1^
*T* = 296 K
0.78 × 0.45 × 0.27 mm

#### Data collection

Stoe IPDS-2 diffractometer
Absorption correction: integration (*X-RED32*; Stoe & Cie, 2002 ▶) *T*_min_ = 0.973, *T*_max_ = 0.989
11187 measured reflections
1672 independent reflections
1554 reflections with *I* > 2σ(*I*)
*R*_int_ = 0.026

#### Refinement

*R*[*F* ^2^ > 2σ(*F* ^2^)] = 0.027
*wR*(*F* ^2^) = 0.074
*S* = 1.07
1672 reflections
192 parameters
1 restraint
H-atom parameters constrained
Δρ_max_ = 0.11 e Å^−3^
Δρ_min_ = −0.10 e Å^−3^

### 

Data collection: *X-AREA* (Stoe & Cie, 2002 ▶); cell refinement: *X-AREA*; data reduction: *X-RED32* (Stoe & Cie, 2002 ▶); program(s) used to solve structure: *SHELXS97* (Sheldrick, 2008 ▶); program(s) used to refine structure: *SHELXL97* (Sheldrick, 2008 ▶); molecular graphics: *ORTEP-3 for Windows* (Farrugia, 1997 ▶); software used to prepare material for publication: *WinGX* (Farrugia, 1999 ▶).

## Supplementary Material

Crystal structure: contains datablocks I, global. DOI: 10.1107/S1600536809009350/hk2635sup1.cif

Structure factors: contains datablocks I. DOI: 10.1107/S1600536809009350/hk2635Isup2.hkl

Additional supplementary materials:  crystallographic information; 3D view; checkCIF report

## Figures and Tables

**Table 1:** Hydrogen-bond geometry (Å, °)

*D*—H⋯*A*	*D*—H	H⋯*A*	*D*⋯*A*	*D*—H⋯*A*
C2—H2⋯O3^i^	0.93	2.60	3.300 (2)	133
C6—H6⋯O2^ii^	0.93	2.44	3.312 (3)	157
C9—H9⋯N1^ii^	0.98	2.55	3.497 (2)	162
C10—H10⋯O5^ii^	0.98	2.66	3.481 (2)	142
C15—H15a⋯O4^iii^	0.96	2.64	3.403 (3)	137

Symmetry codes: (i) 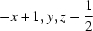; (ii) 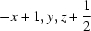; (iii) 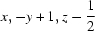.

**Fig. 1.**
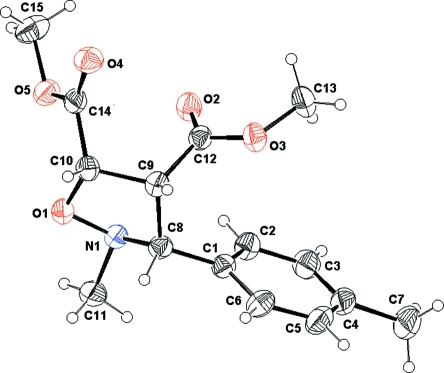
The molecular structure of the title molecule, with the atom-numbering scheme. Displacement ellipsoids are drawn at the 50% probability level.

## supplementary crystallographic information

### Comment

Nitrones are members of a class of compounds which are commonly used as precursors in the syntheses of natural products (Tufariello, 1984), as spin-trapping reagents in the identification of transient radicals (Villamena & Zweier, 2004), and as therapeutic agents (Floyd & Hensley, 2000; Inanami & Kuwabara, 1995) such as in the case of disodium-[(*tert*-butylimino) -methyl]benzene-1,3-disulfonate N-oxide (NXY-059) which is in clinical trials in the USA for the treatment of neurodegenerative disease (Becker *et al.*, 2002). In recent years, it has become clear that reactive oxygen species (ROS) (*e.g.*, radicals: O_2_.^-^, HO., HO_2_., RO_2_., RO., CO_3_.^-^, and CO_2_.^-^; and non-radicals such as H_2_O_2_, HOCl, O_3_, ^1^O_2_, and ROOH) are critical mediators in cardiovascular dysfunction, neurodegenerative diseases, oncogenesis, lung damage and aging, to name a few (Halliwell, 2001*a*; 2001*b*; Zweier & Talukder, 2006). Electron paramagnetic resonance (EPR) spectroscopy has been an indispensable tool for the detection of these ROS *via* spin trapping [Villamena & Zweier, 2004; Janzen, 1971; Janzen,1980; Janzen & Haire, 1990; Villamena *et al.*, 2007). The nitrone-based spin traps, 5,5-dimethyl-1-pyrroline N-oxide (DMPO), 5-diethoxyphosphoryl-5- methyl-pyrroline N-oxide (DEPMPO) and 5-ethoxycarbonyl-5-methyl-pyrroline N-oxide (EMPO), are the most commonly used spin-trapping reagents and have contributed significantly to the understanding of important free radical- mediated processes in chemical, biochemical, and biological systems in spite of their many limitations. The 1,3-dipolar cycloaddition of nitrones and alkenes is a powerful synthetic device that allows up to three new stereogenic centers to be assembled in a stereospecific manner in a single step (Confalone & Huie, 1988; Torssell, 1988; Frederickson, 1997; Gothelf & Jorgensen, 1998). The syntheses of isoxazolidine derivatives is an important subject in organic chemistry because they are found in the structure of most natural compounds and drugs. In recent years, isoxazolidine derivatives have been synthesized in high yield *via* intermolecular cycloaddition of *N*-methylnitrone with disubstituted olefins and are employed for biological evaluation. In view of the importance of the isoxazolidines, we report herein the crystal structure of the title compound.

In the molecule of the title compound (Fig. 1), the bond lengths (Allen *et al.*,  1987) and angles are within normal ranges. Ring A (C1-C6) is, of course, planar, while ring B (O1/N1/C8-C10) adopts envelope conformation with N1 atom displaced by 0.676 (3) Å from the plane of the other ring atoms.

In the crystal structure, weak intermolecular C-H···O and C-H···N hydrogen bonds (Table 1) link the molecules, in which they may be effective in the stabilization of the structure.

### Experimental

N-Methyl-C-(-4-methylphenyl) nitrone, was prepared from 4-methyl benzaldehyde, N-methyl-hydroxylamine hydrochloride and sodium carbonate in CH_2_Cl_2_ according to the literature method (Heaney *et al.*, 2001). For the preparation of the title compound, N-methyl-C-(-4-methylphenyl) nitrone (453 mg, 3 mmol) and dimethylmaleate (475 mg, 3,3 mmol) were dissolved in benzene (50 ml). The reaction mixture was refluxed for 9 h, and monitored by TLC. After evaporation of the solvent, the reaction mixture was separated by column chromatography, using the mixture of hexane/ethyl acetate (1:1) as the eluant. The *cis*-isomer, was recrystallized from CHCl_3_/hexane (1:3) in 2 d (m.p. 371-372 K).

### Refinement

H atoms were positioned geometrically, with C-H = 0.93, 0.98 and 0.96 Å for aromatic, methine and methyl H, respectively, and constrained to ride on their parent atoms, with U_iso_(H) = xU_eq_(C), where x = 1.5 for methyl H and x = 1.2 for all other H atoms. The absolute structure could not be determined reliably, and 1474 Friedel pairs were averaged before the last cycle of refinement.

### Crystal data

**Table:**

C_15_H_19_NO_5_	*F*(000) = 1248
*M**_r_* = 293.31	*D*_x_ = 1.285 Mg m^−^^3^
Orthorhombic, *C**c**c*2	Mo *K*α radiation, λ = 0.71073 Å
Hall symbol: C 2 -2c	Cell parameters from 11187 reflections
*a* = 15.3832 (7) Å	θ = 1.7–28.0°
*b* = 19.7959 (8) Å	µ = 0.10 mm^−^^1^
*c* = 9.9612 (3) Å	*T* = 296 K
*V* = 3033.4 (2) Å^3^	Prism, colorless
*Z* = 8	0.78 × 0.45 × 0.27 mm

### Data collection

**Table:**

Stoe IPDS-2 diffractometer	1672 independent reflections
Radiation source: sealed X-ray tube	1554 reflections with *I* > 2σ(*I*)
plane graphite	*R*_int_ = 0.026
Detector resolution: 6.67 pixels mm^-1^	θ_max_ = 26.5°, θ_min_ = 1.7°
ω scan rotation method	*h* = −18→19
Absorption correction: integration (*X-RED32*; Stoe & Cie, 2002)	*k* = −24→24
*T*_min_ = 0.973, *T*_max_ = 0.989	*l* = −12→12
11187 measured reflections	

### Refinement

**Table:**

Refinement on *F*^2^	Secondary atom site location: difference Fourier map
Least-squares matrix: full	Hydrogen site location: inferred from neighbouring sites
*R*[*F*^2^ > 2σ(*F*^2^)] = 0.027	H-atom parameters constrained
*wR*(*F*^2^) = 0.074	*w* = 1/[σ^2^(*F*_o_^2^) + (0.0483*P*)^2^ + 0.1649*P*] where *P* = (*F*_o_^2^ + 2*F*_c_^2^)/3
*S* = 1.07	(Δ/σ)_max_ = 0.001
1672 reflections	Δρ_max_ = 0.11 e Å^−^^3^
192 parameters	Δρ_min_ = −0.10 e Å^−^^3^
1 restraint	Extinction correction: *SHELXL97* (Sheldrick, 2008), Fc^*^=kFc[1+0.001xFc^2^λ^3^/sin(2θ)]^-1/4^
Primary atom site location: structure-invariant direct methods	Extinction coefficient: 0.0024 (3)

### Special details

**Table:**

Geometry. All e.s.d.'s (except the e.s.d. in the dihedral angle between two l.s. planes) are estimated using the full covariance matrix. The cell e.s.d.'s are taken into account individually in the estimation of e.s.d.'s in distances, angles and torsion angles; correlations between e.s.d.'s in cell parameters are only used when they are defined by crystal symmetry. An approximate (isotropic) treatment of cell e.s.d.'s is used for estimating e.s.d.'s involving l.s. planes.
Refinement. Refinement of *F*^2^ against ALL reflections. The weighted *R*-factor *wR* and goodness of fit *S* are based on *F*^2^, conventional *R*-factors *R* are based on *F*, with *F* set to zero for negative *F*^2^. The threshold expression of *F*^2^ > σ(*F*^2^) is used only for calculating *R*-factors(gt) *etc*. and is not relevant to the choice of reflections for refinement. *R*-factors based on *F*^2^ are statistically about twice as large as those based on *F*, and *R*- factors based on ALL data will be even larger.

### Fractional atomic coordinates and isotropic or equivalent isotropic displacement parameters (Å^2^)

**Table:**

	*x*	*y*	*z*	*U*_iso_*/*U*_eq_	
O1	0.43759 (8)	0.40270 (6)	0.68227 (16)	0.0583 (4)	
O2	0.61162 (9)	0.28606 (7)	0.63405 (15)	0.0577 (3)	
O3	0.60989 (9)	0.22965 (7)	0.82785 (15)	0.0585 (3)	
O4	0.66458 (9)	0.42093 (7)	0.75011 (16)	0.0626 (4)	
O5	0.58517 (8)	0.43642 (7)	0.56403 (14)	0.0578 (3)	
N1	0.41179 (9)	0.33370 (7)	0.64475 (16)	0.0485 (3)	
C1	0.41288 (11)	0.22248 (9)	0.75835 (19)	0.0465 (4)	
C2	0.43264 (13)	0.18797 (10)	0.6418 (2)	0.0555 (4)	
H2	0.4530	0.2114	0.5672	0.067*	
C3	0.42216 (14)	0.11819 (11)	0.6357 (3)	0.0647 (5)	
H3	0.4352	0.0958	0.5561	0.078*	
C4	0.39317 (13)	0.08153 (10)	0.7437 (3)	0.0650 (6)	
C5	0.37474 (15)	0.11654 (12)	0.8606 (3)	0.0680 (6)	
H5	0.3561	0.0928	0.9358	0.082*	
C6	0.38338 (14)	0.18582 (11)	0.8680 (2)	0.0598 (5)	
H6	0.3693	0.2081	0.9472	0.072*	
C7	0.37937 (18)	0.00580 (14)	0.7372 (5)	0.0968 (10)	
H7A	0.3207	−0.0046	0.7626	0.145*	
H7B	0.4189	−0.0162	0.7975	0.145*	
H7C	0.3897	−0.0097	0.6472	0.145*	
C8	0.42456 (11)	0.29783 (9)	0.77189 (18)	0.0460 (4)	
H8	0.3825	0.3149	0.8376	0.055*	
C9	0.51660 (11)	0.32237 (8)	0.81227 (17)	0.0459 (4)	
H9	0.5235	0.3215	0.9101	0.055*	
C10	0.51412 (11)	0.39570 (8)	0.76058 (19)	0.0494 (4)	
H10	0.5071	0.4249	0.8393	0.059*	
C11	0.32106 (12)	0.33992 (12)	0.6060 (3)	0.0650 (5)	
H11A	0.3171	0.3638	0.5223	0.097*	
H11B	0.2900	0.3644	0.6740	0.097*	
H11C	0.2962	0.2957	0.5960	0.097*	
C12	0.58471 (11)	0.27910 (8)	0.74591 (19)	0.0461 (4)	
C13	0.66586 (15)	0.17845 (11)	0.7714 (3)	0.0746 (6)	
H13A	0.6799	0.1458	0.8392	0.112*	
H13B	0.7183	0.1991	0.7392	0.112*	
H13C	0.6366	0.1565	0.6984	0.112*	
C14	0.59637 (11)	0.41814 (8)	0.6902 (2)	0.0475 (4)	
C15	0.66179 (16)	0.46013 (14)	0.4967 (3)	0.0802 (7)	
H15A	0.6475	0.4723	0.4060	0.120*	
H15B	0.7049	0.4250	0.4960	0.120*	
H15C	0.6843	0.4989	0.5429	0.120*	

### Atomic displacement parameters (Å^2^)

**Table:**

	*U*^11^	*U*^22^	*U*^33^	*U*^12^	*U*^13^	*U*^23^
O1	0.0479 (6)	0.0423 (6)	0.0848 (10)	0.0013 (5)	−0.0049 (6)	0.0057 (7)
O2	0.0693 (8)	0.0515 (7)	0.0524 (7)	0.0065 (6)	0.0100 (7)	−0.0007 (6)
O3	0.0641 (8)	0.0521 (7)	0.0592 (8)	0.0095 (6)	−0.0029 (7)	0.0078 (6)
O4	0.0535 (7)	0.0669 (8)	0.0674 (8)	−0.0085 (6)	−0.0091 (6)	−0.0009 (7)
O5	0.0556 (7)	0.0574 (8)	0.0606 (8)	−0.0062 (6)	−0.0025 (6)	0.0096 (6)
N1	0.0461 (7)	0.0452 (7)	0.0541 (9)	−0.0016 (5)	−0.0002 (6)	0.0047 (7)
C1	0.0465 (8)	0.0466 (8)	0.0465 (9)	−0.0038 (6)	−0.0023 (7)	0.0022 (8)
C2	0.0653 (11)	0.0513 (9)	0.0500 (9)	−0.0020 (8)	0.0027 (9)	−0.0002 (9)
C3	0.0672 (11)	0.0546 (11)	0.0724 (13)	−0.0016 (9)	−0.0039 (11)	−0.0135 (11)
C4	0.0526 (10)	0.0491 (10)	0.0935 (16)	−0.0068 (7)	−0.0156 (10)	0.0032 (11)
C5	0.0703 (13)	0.0613 (12)	0.0725 (13)	−0.0159 (10)	−0.0026 (11)	0.0182 (11)
C6	0.0674 (12)	0.0619 (11)	0.0500 (9)	−0.0133 (9)	0.0033 (9)	0.0035 (9)
C7	0.0854 (15)	0.0517 (11)	0.153 (3)	−0.0127 (11)	−0.0152 (19)	−0.0036 (16)
C8	0.0474 (8)	0.0463 (9)	0.0442 (8)	−0.0017 (6)	0.0061 (7)	−0.0014 (7)
C9	0.0532 (9)	0.0448 (8)	0.0398 (8)	−0.0009 (7)	0.0006 (7)	−0.0031 (7)
C10	0.0517 (9)	0.0423 (8)	0.0541 (9)	0.0011 (6)	0.0027 (8)	−0.0073 (8)
C11	0.0483 (9)	0.0661 (12)	0.0806 (14)	−0.0008 (8)	−0.0074 (10)	0.0108 (10)
C12	0.0479 (8)	0.0416 (8)	0.0489 (9)	−0.0023 (6)	−0.0026 (8)	−0.0011 (8)
C13	0.0717 (13)	0.0527 (11)	0.0995 (18)	0.0172 (9)	−0.0032 (13)	0.0027 (12)
C14	0.0491 (9)	0.0384 (7)	0.0549 (9)	−0.0022 (6)	−0.0027 (8)	−0.0046 (8)
C15	0.0757 (14)	0.0924 (16)	0.0726 (15)	−0.0240 (12)	0.0079 (12)	0.0166 (14)

### Geometric parameters (Å, °)

**Table:**

C1—C2	1.381 (3)	C10—O1	1.419 (2)
C1—C6	1.387 (3)	C10—C14	1.513 (3)
C1—C8	1.508 (2)	C10—H10	0.9800
C2—C3	1.392 (3)	C11—N1	1.453 (2)
C2—H2	0.9300	C11—H11A	0.9600
C3—C4	1.373 (4)	C11—H11B	0.9600
C3—H3	0.9300	C11—H11C	0.9600
C4—C5	1.384 (4)	C12—O2	1.197 (2)
C4—C7	1.515 (3)	C12—O3	1.332 (2)
C5—C6	1.380 (3)	C13—O3	1.444 (3)
C5—H5	0.9300	C13—H13A	0.9600
C6—H6	0.9300	C13—H13B	0.9600
C7—H7A	0.9600	C13—H13C	0.9600
C7—H7B	0.9600	C14—O4	1.209 (2)
C7—H7C	0.9600	C14—O5	1.319 (2)
C8—N1	1.465 (2)	C15—O5	1.435 (3)
C8—C9	1.550 (2)	C15—H15A	0.9600
C8—H8	0.9800	C15—H15B	0.9600
C9—C12	1.506 (2)	C15—H15C	0.9600
C9—C10	1.541 (2)	N1—O1	1.4707 (19)
C9—H9	0.9800		
			
C2—C1—C6	118.35 (17)	O1—C10—C14	114.22 (15)
C2—C1—C8	122.56 (16)	O1—C10—C9	107.23 (13)
C6—C1—C8	119.07 (17)	C14—C10—C9	114.25 (13)
C1—C2—C3	120.1 (2)	O1—C10—H10	106.9
C1—C2—H2	119.9	C14—C10—H10	106.9
C3—C2—H2	119.9	C9—C10—H10	106.9
C4—C3—C2	121.9 (2)	N1—C11—H11A	109.5
C4—C3—H3	119.1	N1—C11—H11B	109.5
C2—C3—H3	119.1	H11A—C11—H11B	109.5
C3—C4—C5	117.47 (18)	N1—C11—H11C	109.5
C3—C4—C7	122.3 (3)	H11A—C11—H11C	109.5
C5—C4—C7	120.2 (3)	H11B—C11—H11C	109.5
C6—C5—C4	121.5 (2)	O2—C12—O3	123.68 (17)
C6—C5—H5	119.2	O2—C12—C9	125.72 (17)
C4—C5—H5	119.2	O3—C12—C9	110.57 (16)
C5—C6—C1	120.6 (2)	O3—C13—H13A	109.5
C5—C6—H6	119.7	O3—C13—H13B	109.5
C1—C6—H6	119.7	H13A—C13—H13B	109.5
C4—C7—H7A	109.5	O3—C13—H13C	109.5
C4—C7—H7B	109.5	H13A—C13—H13C	109.5
H7A—C7—H7B	109.5	H13B—C13—H13C	109.5
C4—C7—H7C	109.5	O4—C14—O5	124.86 (18)
H7A—C7—H7C	109.5	O4—C14—C10	120.68 (18)
H7B—C7—H7C	109.5	O5—C14—C10	114.39 (15)
N1—C8—C1	112.71 (14)	O5—C15—H15A	109.5
N1—C8—C9	101.23 (13)	O5—C15—H15B	109.5
C1—C8—C9	116.23 (14)	H15A—C15—H15B	109.5
N1—C8—H8	108.8	O5—C15—H15C	109.5
C1—C8—H8	108.8	H15A—C15—H15C	109.5
C9—C8—H8	108.8	H15B—C15—H15C	109.5
C12—C9—C10	113.98 (14)	C11—N1—C8	113.54 (15)
C12—C9—C8	110.07 (13)	C11—N1—O1	104.36 (14)
C10—C9—C8	100.72 (13)	C8—N1—O1	101.21 (13)
C12—C9—H9	110.6	C10—O1—N1	105.84 (11)
C10—C9—H9	110.6	C12—O3—C13	116.79 (18)
C8—C9—H9	110.6	C14—O5—C15	115.34 (17)
			
C6—C1—C2—C3	0.5 (3)	C8—C9—C10—C14	−137.37 (16)
C8—C1—C2—C3	178.76 (17)	C10—C9—C12—O2	−29.1 (3)
C1—C2—C3—C4	−0.7 (3)	C8—C9—C12—O2	83.2 (2)
C2—C3—C4—C5	−0.2 (3)	C10—C9—C12—O3	152.90 (14)
C2—C3—C4—C7	178.4 (2)	C8—C9—C12—O3	−94.81 (17)
C3—C4—C5—C6	1.3 (3)	O1—C10—C14—O4	172.80 (15)
C7—C4—C5—C6	−177.4 (2)	C9—C10—C14—O4	−63.2 (2)
C4—C5—C6—C1	−1.4 (3)	O1—C10—C14—O5	−4.3 (2)
C2—C1—C6—C5	0.5 (3)	C9—C10—C14—O5	119.63 (16)
C8—C1—C6—C5	−177.80 (18)	C1—C8—N1—C11	75.72 (19)
C2—C1—C8—N1	31.2 (2)	C9—C8—N1—C11	−159.42 (16)
C6—C1—C8—N1	−150.60 (17)	C1—C8—N1—O1	−173.05 (13)
C2—C1—C8—C9	−85.0 (2)	C9—C8—N1—O1	−48.19 (14)
C6—C1—C8—C9	93.2 (2)	C14—C10—O1—N1	107.82 (15)
N1—C8—C9—C12	−85.19 (15)	C9—C10—O1—N1	−19.84 (17)
C1—C8—C9—C12	37.3 (2)	C11—N1—O1—C10	161.41 (16)
N1—C8—C9—C10	35.45 (15)	C8—N1—O1—C10	43.31 (16)
C1—C8—C9—C10	157.90 (15)	O2—C12—O3—C13	−6.6 (3)
C12—C9—C10—O1	108.09 (17)	C9—C12—O3—C13	171.52 (16)
C8—C9—C10—O1	−9.72 (16)	O4—C14—O5—C15	0.9 (3)
C12—C9—C10—C14	−19.6 (2)	C10—C14—O5—C15	177.92 (18)

### Hydrogen-bond geometry (Å, °)

**Table:**

*D*—H···*A*	*D*—H	H···*A*	*D*···*A*	*D*—H···*A*
C2—H2···O3^i^	0.93	2.60	3.300 (2)	133
C6—H6···O2^ii^	0.93	2.44	3.312 (3)	157
C9—H9···N1^ii^	0.98	2.55	3.497 (2)	162
C10—H10···O5^ii^	0.98	2.66	3.481 (2)	142
C15—H15a···O4^iii^	0.96	2.64	3.403 (3)	137

Symmetry codes: (i) −*x*+1, *y*, *z*−1/2; (ii) −*x*+1, *y*, *z*+1/2; (iii) *x*, −*y*+1, *z*−1/2.
